# Microvascular obstruction by delayed contrast-enhanced MRI remains a strong predictor of left ventricular remodeling even after successful reperfusion in acute myocardial infarction

**DOI:** 10.1186/1532-429X-17-S1-P167

**Published:** 2015-02-03

**Authors:** Lin Zhang, Olivier Huttin, Nicolas Girerd, Christian de Chillou, Pierre-Yves Marie, Freddy Odille, Jacques Felblinger, Damien Mandry

**Affiliations:** INSERM U947, IADI, University of Lorraine, Nancy, France; Department of Cardiology, CHU Nancy, Nancy, France; INSERM, CIC-P, Nancy, France; Department of Radiology, CHU Nancy, Nancy, France; INSERM, U961, University of Lorraine, Nancy, France

## Background

Microvascular obstruction (MVO) after coronary reperfusion for acute myocardial infarction (AMI) implies advanced myocardial damage and is recognized as a negative prognostic value. Delayed contrast-enhanced MRI is the preferred MR imaging technique for evaluation of MVO. In this study, we assessed the influence of MVO on post-infarction left ventricular (LV) remodeling and dysfunction.

## Methods

92 successfully reperfused ST-segment elevation MI patients were prospectively studied. Cardiovascular magnetic resonance was performed at 2±4 days (baseline) and 6 months (follow-up) on a 3-T scanner (Signa HDxt, GE Medical System), including cine SSFP and delayed contrast-enhanced sequences. Contrast-enhanced images were acquired 10-20 minutes after gadolinium injection (0.1mmol/kg; Dotarem®, GUERBET, France). LV volumes (end-diastolic/end-systolic volume, LVEDV/LVESV), ejection fraction (LVEF), infarct size and MVO (defined as a dark zone within the infarcted segments) were analyzed using MASS Analytical Software (Leiden University Medical Center, Dept. Radiology, Division of Image Processing). MVO and infarct size were expressed as percentage of LV mass (%LV). Adverse LV remodeling was defined as an increase in LV end-diastolic volume ≥20% at follow-up. Data from patients with MVO and those without MVO were compared. Student t-test and Fisher's exact test were used for continuous data and categorical data comparison, respectively. A level of p<0.05 was considered statistically significant.

## Results

Even though successful reperfusion (TIMI flow grade 3) was achieved, MVO was still detected in 62% (57/92) patients at baseline (size: 6.6±5.7, %LV). Comparison data were shown in Table 1. Infarct size diminished significantly regardless of the presence or absence of MVO. Patients with MVO had significantly larger infarct size and lower LV ejection fraction (LVEF) both at baseline and follow-up (*p*<0.05 for all). In addition, although LVEF improved equally in two groups (*p*>0.05), there was a significant increase of LVEDV in MVO group (13.8±23.7mL vs. -0.13±16.6mL, *p*<0.05). Furthermore, adverse LV remodeling, using the predefined criterion, occurred in 13 out of 57 patients with MVO and in only 2 out of 35 patients without MVO (Fisher's test: *p*=0.04). Besides, reduction of LVESV, likely related to the regression of stunning, was much higher in no MVO group (*p*<0.05).

**Table 1:** Data comparison between patients with MVO and without MVO

Variables	With MVO (N=57)		Without MVO (N=35)	
	**Baseline**	**Follow-up**	**Baseline**	**Follow-up**
Infarct size, %LV	38.3±10.5^§^	29.5±10.5^*#^	30.3±11.0	22.7±9.8^*^
LVEF, %	40.4±7.6^§^	46.7±7.6^*#^	44.3±6.5	51.9±7.3^*^
LVEDV, mL	185.2±32.9	199.0±34.4^*#^	170.2±32.4	170.1±30.2
LVESV, mL	111.1±27.3^§^	107.3±30.1^#^	94.5±19.6	81.8±19.3^*^
∆LVEF, %	6.3±6.3		7.6±5.9	
∆LVEDV, mL	13.8±23.7		-0.13±16.6^†^	
∆LVEDV, %	8.3±12.8		0.76±10.6^†^	
∆LVESV, mL	-3.8±20.8		-12.7±11.8^†^	
∆LVESV, %	-2.7±16.6		-13.0±12.9^†^	

Note. - Data are expressed as Mean ± SD. EF=ejection fraction; EDV=end-diastolic volume; ESV=end-systolic volume; ∆LVEF indicates absolute change from baseline to follow-up while ∆LVED(S)V indicates absolute change (mL) and relative change (%).

^*^p<0.05 for comparison between baseline and follow-up in two groups.

^§^p<0.05, ^#^p<0.05 for comparison of baseline and follow-up data between patients with and without MVO, respectively.

^†^p<0.05 for comparison of changes in two groups.

## Conclusions

Patients with MVO experienced more pronounced LV remodeling and dysfunction even under the condition of restoration of epicardial coronary flow. The negative prognostic value of MVO helps patient risk stratification and might be a promising surrogate endpoint to evaluate reperfusion therapy strategies.

## Funding

Financical support from Guerbet company.

